# Gait Progression Over 6 Years in Parkinson’s Disease: Effects of Age, Medication, and Pathology

**DOI:** 10.3389/fnagi.2020.577435

**Published:** 2020-10-15

**Authors:** Joanna Wilson, Lisa Alcock, Alison J. Yarnall, Sue Lord, Rachael A. Lawson, Rosie Morris, John-Paul Taylor, David J. Burn, Lynn Rochester, Brook Galna

**Affiliations:** ^1^Translational and Clinical Research Institute, Faculty of Medical Sciences, Newcastle University, Newcastle upon Tyne, United Kingdom; ^2^The Newcastle upon Tyne NHS Foundation Trust, Newcastle upon Tyne, United Kingdom; ^3^Auckland University of Technology, Auckland, New Zealand; ^4^Department of Sport, Exercise and Rehabilitation, Northumbria University, Newcastle upon Tyne, United Kingdom; ^5^Faculty of Medical Sciences, Newcastle University, Newcastle upon Tyne, United Kingdom; ^6^School of Biomedical, Nutritional and Sport Sciences, Newcastle University, Newcastle upon Tyne, United Kingdom

**Keywords:** gait, walking, Parkinson’s disease, neurological disorders, aging, longitudinal

## Abstract

**Background**: Gait disturbance is an early, cardinal feature of Parkinson’s disease (PD) associated with falls and reduced physical activity. Progression of gait impairment in Parkinson’s disease is not well characterized and a better understanding is imperative to mitigate impairment. Subtle gait impairments progress in early disease despite optimal dopaminergic medication. Evaluating gait disturbances over longer periods, accounting for typical aging and dopaminergic medication changes, will enable a better understanding of gait changes and inform targeted therapies for early disease. This study aimed to describe gait progression over the first 6 years of PD by delineating changes associated with aging, medication, and pathology.

**Methods**: One-hundred and nine newly diagnosed PD participants and 130 controls completed at least two gait assessments. Gait was assessed at 18-month intervals for up to 6 years using an instrumented walkway to measure sixteen spatiotemporal gait characteristics. Linear mixed-effects models assessed progression.

**Results**: Ten gait characteristics significantly progressed in PD, with changes in four of these characteristics attributable to disease progression. Age-related changes also contributed to gait progression; changes in another two characteristics reflected both aging and disease progression. Gait impairment progressed irrespective of dopaminergic medication change for all characteristics except step width variability.

**Conclusions**: Discrete gait impairments continue to progress in PD over 6 years, reflecting a combination of, and potential interaction between, disease-specific progression and age-related change. Gait changes were mostly unrelated to dopaminergic medication adjustments, highlighting limitations of current dopaminergic therapy and the need to improve interventions targeting gait decline.

## Introduction

Gait impairment is a common and debilitating feature of Parkinson’s disease (PD) which manifests in the early and even prodromal disease stages (Galna et al., 2015; Del Din et al., 2019a). It impacts independence and quality of life (Curtze et al., 2016) and is associated with an increased falls risk (Lord et al., 2016) and reduced physical activity levels (Lord et al., 2013c; Del Din et al., 2019b). Dopaminergic medication can provide immediate improvements in some characteristics of PD gait including walking speed (Bryant et al., 2011; Sterling et al., 2015). However, other characteristics such as gait variability continue to worsen over time despite optimal dopaminergic treatment (Galna et al., 2015). This suggests that additional interventions are required to mitigate against gait impairment and its progression (Lord et al., 2011) to reduce falls and increase physical activity.

Developing successful interventions is made difficult by the multifaceted etiology of gait impairment in PD. Gait impairment is underpinned by a complex interaction of pathology, age-related changes, compensatory mechanisms and, eventually, secondary deconditioning due to restricted mobility (Lord et al., 2013a). This explains, in part, the highly variable response to dopaminergic medication. Teasing apart changes in gait due to aging, PD, and medication is a fundamental step towards developing therapies that minimize gait impairment.

Previous work (Rochester et al., 2017) showed that discrete gait characteristics (variability of step time, step length, and step width) progress more rapidly over 3 years in a newly diagnosed, optimally medicated PD cohort than in age-matched controls. This provided the first evidence suggesting that the progression rate is driven by several factors related to age and pathology. Other studies have identified a shortening of step length and swing time over 18 months from PD diagnosis (Galna et al., 2015) and greater increases in step or stride time variability over 5 years in early and moderate stages of PD compared to healthy controls (Hobert et al., 2019; Micó-Amigo et al., 2019). However, relatively short study timeframes, inconsistent reporting of gait characteristics, lack of control cohorts, limited exploration of medication effects, and wide variation in disease duration at inclusion have limited insights into progression, particularly concerning background aging and medication.

To comprehensively determine the changes in gait which are specifically due to PD progression, gait change should be modeled over a longer timeframe concerning an age-matched control cohort and with consideration of changes in dopaminergic medication. Additionally, modeling gait progression from diagnosis enables a more precise understanding of gait changes occurring before the onset of more severe motor symptoms and, therefore, during a time when interventions may be most beneficial.

The aims of this study were therefore to: (i) identify gait characteristics that significantly changed over 6 years in newly diagnosed PD and healthy age-matched controls; (ii) evaluate gait changes in the PD cohort which related to aging and disease progression by comparing rates of gait change between PD and control groups; and (iii) explore the relationship between gait changes and changes in dopaminergic medication dose in early PD. It was hypothesized that:

(1) Variability of step time, step length, and step width will significantly change over the first 6 years of PD, as they did over the first 3 years (Rochester et al., 2017).
(2) Additional characteristics to those already identified in our earlier work (Rochester et al., 2017) will demonstrate significant change over 6 years that are specific to the PD cohort, to reflect the additional progression of PD pathology over a longer timeframe as well as any potential contributions of compensatory mechanisms and secondary deconditioning due to restricted mobility.
(3) Change in selective gait characteristics such as step length will be associated with changes in dopaminergic medication, as have been identified previously (Rochester et al., 2017), to indicate dopa-responsive and dopa-resistant gait characteristics.

To address these hypotheses, we have extended on our previous 36-month longitudinal analysis of gait in an incident cohort of people with PD (tested “on” their medication) and age-matched controls (Rochester et al., 2017), to now include more recent data from 54 and 72 months follow up assessments.

## Materials and Methods

### Participants

Participants with newly diagnosed idiopathic PD were recruited into ICICLE-GAIT, a study nested within the ICICLE-PD study (Incidence of Cognitive Impairment in Cohorts with Longitudinal Evaluation—PD). Recruitment was conducted between June 2009 and December 2011, as described fully in previous publications (Khoo et al., 2013; Lord et al., 2014; Yarnall et al., 2014). Briefly, people with PD had to be diagnosed (and confirmed at each follow-up assessment) with idiopathic PD according to the UK Parkinson’s Disease Brain Bank criteria by a movement disorders specialist and were excluded if they presented with significant memory impairment [Mini-Mental State Exam (MMSE) <24], dementia with Lewy bodies, drug-induced parkinsonism, “vascular” parkinsonism, progressive supranuclear palsy, multiple system atrophy, cortico-basal degeneration or poor command of English. Potential participants were excluded if they presented with any neurological (other than PD), orthopedic, or cardiothoracic conditions that may have severely affected their walking or safety during the testing sessions. Also, control participants had to be at least 60 years of age, able to walk independently without a walking aid, and have no significant cognitive impairment, mood, or movement disorder.

**Figure 1:**
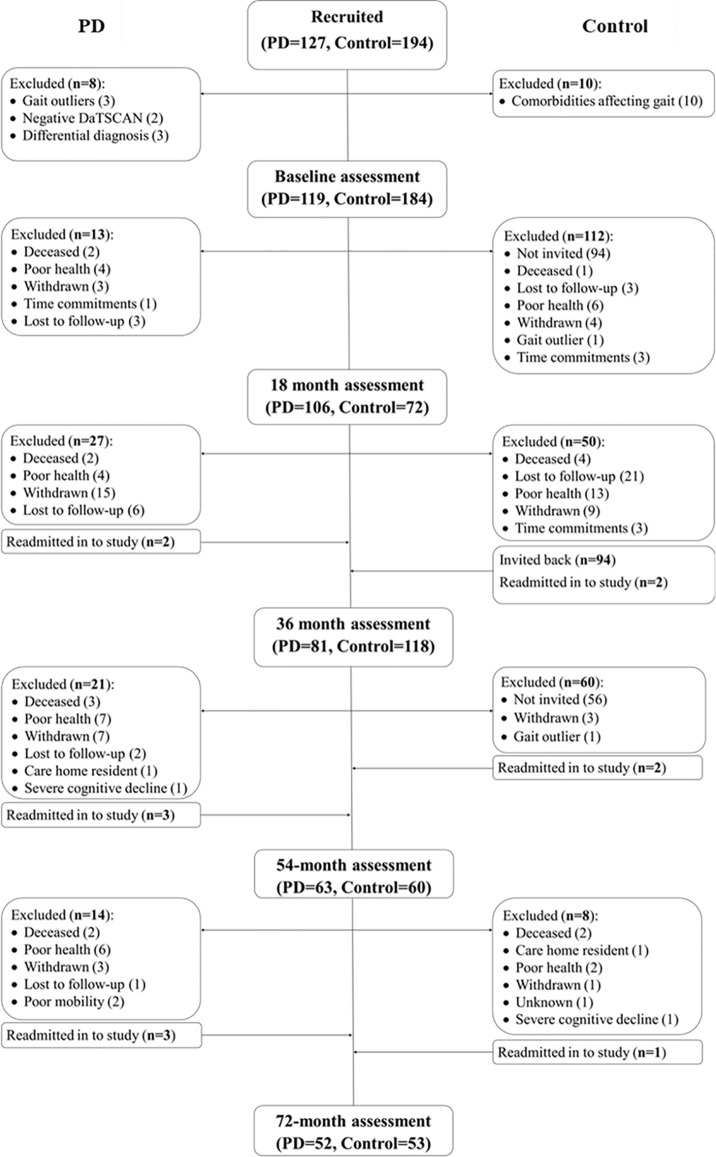
Flow chart indicating participant recruitment and attrition throughout the ICICLE-GAIT study. Some participants were readmitted into the study having previously withdrawn due to acute pain or illness.

Assessments were completed every 18-months from baseline recruitment up to 72-months after baseline. Participant recruitment and attrition are displayed in Figure 1. The present analyses included an additional participant compared to previous work from the ICICLE-GAIT study (Rochester et al., 2017), due to the participant re-entering the study at the 54-month assessment following acute orthopedic issues. PD participants were tested “on” medication, defined as 1 h after PD medication. Age-matched controls were recruited from the community to evaluate changes due to typical aging. The study was approved by the Newcastle and North Tyneside Research Ethics Committee and participants gave written informed consent.

### Gait Assessment

Participants walked at their self-selected pace for 2 min around a 25-m oval circuit which included a 7-m long instrumented walkway (240 Hz sampling frequency, Platinum model GAITRite, software version 4.5, CIR Systems, USA). At least 40 steps were completed over the walkway per participant to ensure robust measurement of gait variability (Galna et al., 2013). Gait outcomes were derived and quantified according to an *a priori* model developed for older adults (Lord et al., 2013b), and validated in PD (Lord et al., 2013a), that describes 16 discrete gait characteristics within five domains (Pace; Variability; Rhythm; Asymmetry; and Postural control—see Table 2 for a list of characteristics; Galna et al., 2015). Methods to calculate the gait characteristics have been detailed previously (Galna et al., 2013) but are briefly detailed here:

Step length: distance in the direction of travel from the heel during foot contact to the contralateral heel during the next foot contact.

Step time: duration from foot contact to contralateral foot contact.

Step velocity: step length divided by step time.

Stance time: duration from foot contact to when the foot is lifted off the ground.

Swing time: duration from when the foot is lifted off the ground to its next contact with the ground.

Step width: the perpendicular distance between the heel during foot contact and the direction of travel of the contralateral foot.

The mean, variability, and asymmetry of gait characteristics were calculated as follows:

Mean: the mean of left and right steps were calculated separately before calculating the (overall) mean of the left and right steps.

Variability: the variance of the left and right steps were calculated separately before calculating the overall variability as the square root of the average (of left and right) variance, resulting in a combined standard deviation.

Asymmetry: the mean of left and right steps were calculated separately before calculating the absolute (non-directional) difference between the means of left and right steps.

**Table 1:** Baseline demographic characteristics.

Characteristic	Control (*n* = 130)	PD (*n* = 109)	Group difference (test statistic, *p*)
Age (years)	69.5 (7.4)	67.4 (9.9)	*t* = 1.87, *p* = 0.063
Sex	55% f (72 f, 58 m)	34% f (37 f, 72 m)	*χ*^2^ = 10.99, *p* = 0.001*
Height (m)	1.68 (0.10)	1.70 (0.08)	*t* = −1.05, *p* = 0.294
Body mass (kg)	78.3 (14.9)	78.8 (15.4)	*t* = −0.28, *p* = 0.782
GDS-15	1.3 (2.0)	2.6 (2.2)	U = 4,077, *p* < 0.001*
Education (years)	14 (4)	13 (4)	*U* = 3,723, *p* = 0.373
NART	117 (8)	115 (11)	*U* = 6,650, *p* = 0.483
MoCA^⊢^	27 (2)	25 (4)	U = 2,601, *p* ≤ 0.001*
MMSE	29 (1)	29 (1)	U = 5,047, *p* ≤ 0.001*
Sit to stand (s)	12.1 (3.4)	14.1 (4.7)	U = 4,841, *p* ≤ 0.001*
Single leg stance (s)	16.2 (11.4)	13.8 (10.8)	*U* = 6,382, *p* = 0.185
ABC (0-100)	92.7 (9.5)	82.2 (19.5)	U = 4,880, *p* ≤ 0.001*
MDS-UPDRS III (0–132)	-	25.0 (10.3)	-
H&Y stage *n* (%)	-	I 26 (23.9%); II 65 (59.6%); III 18 (16.5%)	-
*n* (%) who report FOG	-	11 (10.1%)	-
LEDD (mg/day)	-	175 (132)	-
ADS (total summed score)	-	0.7 (1.3)	-

Notes: BMI, Body Mass Index; GDS-15, Geriatric Depression Scale; NART, National Adult Reading Test; MoCA, Montreal Cognitive Assessment; MMSE, Mini-Mental State Examination; ABC, Activities-specific Balance Confidence; MDS-UPDRS III, Movement Disorders Society Unified Parkinson’s Disease Rating Scale; H&Y, Hoehn& Yahr; FOG, Freezing of Gait; LEDD, Levodopa Equivalent Daily Dose; ADS, Anticholinergic Drug Scale. ^⊢^indicates a smaller sample size; - indicates PD specific metrics not collected for control participants; control *n* = 73, PD *n* = 104. *p < 0.05.

**Table 2:** Modeled change in gait characteristics over 72-months for control and Parkinson’s disease (PD) participants.

		Controls			PD		Group × Time interaction			LEDD × Time interaction		
	Δ per year	*p*	95% CI	Δ per year	*p*	95% CI	β	*P*	95% CI	β	*p*	95% CI
Pace
Step velocity (m/s)	−0.0053	0.046*	−0.0105, −0.0002	−0.0124	0.004*	−0.0208, −0.0042	−0.0068	0.158	−0.0163, 0.0026	7.4 × e^−6^	0.415	−10.4 × e^−6^, 25.2 × e^−6^
Step length (m)	−0.0047	<0.001*	−0.0068, −0.0026	−0.0092	<0.001*	−0.0131, −0.0054	−0.0044	0.035*	−0.0085, −0.0003	−0.7 × e^−6^	0.855	−7.7 × e^−6^, 6.4 × e^−6^
Swing time sd (ms)	0.0113	0.893	−0.1583, 0.1767	0.3968	0.016*	0.0752, 0.7239	0.3755	0.031*	0.0345, 0.7187	−0.6 × e^−3^	0.301	−1.7 × e^−3^, 0.5 × e^−3^
Variability												
Step time sd (ms)	−0.0265	0.773	−0.2066, 0.1559	0.5131	0.007*	0.1491, 0.8958	0.5198	0.009*	0.1320, 0.9129	−0.3 × e^−3^	0.535	−1.4 × e^−3^, 0.7 × e^−3^
Stance time sd (ms)	−0.0041	0.976	−0.2699, 0.2620	0.4574	0.061	−0.0199, 0.9461	0.4826	0.075	−0.0486, 1.0187	−0.9 × e^−3^	0.283	−2.4 × e^−3^, 0.7 × e^−3^
Step velocity sd (m/s)	−0.0001	0.708	−0.0006, 0.0004	0.0007	0.107	−0.0001, 0.0016	0.0009	0.060	−0.00003, 0.0019	0.01 × e^−6^	0.993	−2.6 × e^−6^, 2.6 × e^−6^
Step length sd (m)	0.0003	0.002*	0.0001, 0.0005	0.0009	<0.001*	0.0005, 0.0013	0.0006	0.004*	0.0002, 0.0010	−0.2 × e^−6^	0.759	−1.4 × e^−6^, 1.1 × e^−6^
Rhythm												
Step time (ms)	−0.9841	0.160	−2.369, 0.3882	−1.789	**0.007**	−3.049, −0.529	−0.8069	0.353	−2.503, 0.8897	−2.1 × e^−3^	0.363	−6.6 × e^−3^, 2.5 × e^−3^
Swing time (ms)	−0.9276	0.006*	−1.579, −0.287	−2.039	<0.001*	−3.095, −0.992	−1.1013	0.068	−2.2846, 0.0831	−0.7 × e^−3^	0.669	−4.3 × e^−3^, 2.8 × e^−3^
Stance time (ms)	−0.9378	0.418	−3.231, 1.342	−1.385	0.183	−3.418, 0.694	−0.3487	0.806	−3.131, 2.462	−4.0 × e^−3^	0.235	−10.7 × e^−3^, 2.7 × e^−3^
Asymmetry												
Step time asy (ms)	0.4435	0.020*	0.0731, 0.8213	−0.6508	0.239	−1.736, 0.451	−1.0796	0.054	−2.176, 0.0238	−0.8 × e^−3^	0.556	−3.7 × e^−3^, 2.0 × e^−3^
Swing time asy (ms)	0.2557	0.069	−0.0202, 0.5304	−0.8137	0.042*	−1.594, −0.027	−1.0142	0.012*	−1.803, −0.2214	−0.4 × e^−3^	0.704	−2.4 × e^−3^, 1.6 × e^−3^
Stance time asy (ms)	0.2213	0.145	−0.0786, 0.5190	−0.7502	0.052	−1.505, 0.011	−0.8782	0.027*	−1.653, −0.0997	−0.6 × e^−3^	0.564	−2.6 × e^−3^, 1.5 × e^−3^
ostural Control												
Step length asy (m)	0.0008	0.025*	−0.0001, −0.0014	0.0009	0.125	−0.0002, −0.0020	0.0002	0.813	−0.0011, 0.0014	0.1 × e^−6^	0.959	−3.0 × e^−6^, 3.1 × e^−6^
Step width (m)	0.0007	0.027*	0.0001, 0.0013	0.0009	0.014*	0.0002, 0.0017	0.0002	0.650	−0.0007, 0.0012	0.6 × e^−6^	0.632	−1.8 × e^−6^, 2.9 × e^−6^
Step width sd (m)	−0.0001	0.290	−0.0003, 0.0001	0.0008	<0.001*	0.0004, 0.0012	0.0009	<0.001*	0.0005, 0.0013	1.6 × e^−6^	<0.001*	0.7 × e^−6^, 2.5 × e^−6^

Notes All models were adjusted for baseline age and sex. asy, asymmetry; sd, standard deviation (gait variability). *p < 0.05.

### Clinical Assessments

Details of the clinical assessments have been described previously (Khoo et al., 2013; Yarnall et al., 2014). Briefly, age, sex, height, mass, and depression [Geriatric Depression Scale (GDS-15)] were recorded. The National Adult Reading Test (NART) assessed premorbid intelligence at baseline. Global cognition was assessed through the Montreal Cognitive Assessment (MoCA) as well as the Mini-Mental State Examination (MMSE). The five times sit-to-stand test was used as a surrogate marker of strength, and confidence in balance during activities of daily living was measured with the Activities-specific Balance Confidence (ABC) scale and the time participants were able to stand on one leg (mean of left and right; capped at 30 s). PD specific motor severity was measured using the Movement Disorders Society Unified Parkinson’s Disease Rating Scale (MDS-UPDRS III) from which the Hoehn and Yahr Stage (H&Y) was assessed. The presence of freezing of gait (FOG) was assessed with the FOG questionnaire. Levodopa equivalent daily dose (LEDD) was calculated at each assessment (Tomlinson et al., 2010). The anticholinergic burden was assessed using the Anticholinergic Drug Scale (ADS), where each medication was classified on a scale from 0 to 3 according to no (0), mild (1), moderate (2), or high (3) anticholinergic activity (Carnahan et al., 2006). Medications not specified on the ADS were reviewed according to the literature to ensure accurate scoring.

### Statistical Analysis

The analysis was completed using SPSS (IBM Corp. V.24, USA) and R (R Foundation for Statistical Computing, V3.5.2, Austria). First, univariate analyses described demographic, clinical, and gait data for both groups at baseline assessment, through Student’s *t*-test, Mann–Whitney *U*, and Chi-square tests as appropriate. The distributions of continuous variables were assessed through the Skewness–Kurtosis test and inspection of boxplots and histograms. Due to high levels of skewness, gait asymmetry data were square-root transformed and temporal variability data were natural log-transformed for cross-sectional analysis; no outliers were removed. Within-group baseline differences between participants who did and did not complete the 72-month gait assessment were also assessed.

Second, linear mixed-effects models (LMEM; R, “lme4” and “lmerTest”) modeled change in each gait characteristic, as these can give appropriate estimates of regression coefficients despite significant participant drop-out across the study period (Little, 1995; Figure 1). Random slope models gave each participant a unique intercept and slope, accounting for individual variability and allowing for correlation between intercept and slope. Even though it is possible to calculate change scores for participants with only one assessment using LMEM, we chose to include only participants who were assessed at least twice to ensure more robust estimates of change in gait. Baseline age and sex were included as fixed effects in all models. Likelihood ratio tests assessed model fit. We performed a preliminary analysis to check for a non-linear change of gait by comparing the fit of a linear model and a model with an added a quadratic term of time for each gait characteristic. The two models were compared using likelihood ratio tests with a *X*^2^ distribution. This analysis indicated there was little evidence (*p* > 0.05) that the quadratic term improved the model fit for any gait characteristic and so a model of linear change over time was used.

There were three stages to longitudinal analyses addressing each of our aims in turn: (i) to quantify the progression of gait impairment over time, basic models independently assessed changes in gait for PD and control groups; (ii), to compare the rate of gait change between the groups, models included the interaction between group and time (Group × Time); and (iii) to evaluate the association between change in gait and change in LEDD, PD models included the interaction between LEDD and time (LEDD × Time).

To aid data interpretation concerning our study aims, significant gait changes in the PD cohort that were related to aging and disease progression were discerned using the following criteria:

(1) Gait-change related only to disease progression: a significant gait change was identified in the PD cohort but not in controls, where the rate of change differed significantly between groups.
(2) Gait change related to aging: a significant gait change was identified in both PD and control cohorts, where the rate of change did not differ significantly between PD and control groups.
(3) Gait change related to both aging and disease progression: a significant gait change in both PD and control cohorts, where the rate of change differed significantly between groups.

Also, individual trajectories of gait change over time were plotted to illustrate variability within the cohorts. We used a threshold of *p* < 0.05 to guide statistical interpretation. *p*-values were not adjusted for multiple comparisons. This allowed direct comparisons with our previous analysis quantifying gait change over the first 3 years of PD (Rochester et al., 2017) and also reduced the risk of type II error. As multiple comparisons increase the risk of type I statistical error, we present full *p*-values for the reader to assess the statistical robustness of our findings.

## Results

### Study Participants and Baseline Assessments

One-hundred and thirty control and 109 PD participants completed at least two assessments, so were included in this analysis (Supplementary Table 1). At baseline, PD participants had similar age, height, mass, and NART scores to controls (Table 1). The PD group had proportionately more males, poorer global cognition (MoCA and MMSE), lower mood (GDS-15), poorer balance confidence (ABC), and were slower performing the sit-to-stand test (*p* ≤ 0.001). The mean time to baseline assessment from PD diagnosis was 6 months, and from the first subjective motor symptom was 27 months. Motor score severity at baseline was low (mean MDS-UPDRS III = 25); 10% had already experienced FOG and most (90%) were taking dopaminergic medication, in keeping with clinical practice. Thirty-one percent of people with PD were taking medication with an anticholinergic burden although the overall burden was low, with a mean score of 0.7, which may reflect increased clinician awareness of the adverse outcomes associated with anticholinergic in PD. For both groups, participants who completed the 72-month assessment (“completers”) were younger and had better baseline performance on the MoCA compared to “non-completers” who did not attend a 72-month assessment (Supplementary Table 2). Additionally, PD completers had better balance (higher ABC and single leg stand time) and less severe motor disease (lower MDS-UPDRS III and H&Y stage) at baseline than non-completers.

Baseline gait characteristics for PD and control groups have previously been detailed (Galna et al., 2015; Rochester et al., 2017). Briefly, 13 of 16 characteristics differed between the groups (Supplementary Table 3); PD participants walked slower with a shorter step length, longer step and stance time, and greater asymmetry and variability (except for less step width variability) compared to controls. PD completers walked significantly faster with a longer step at baseline compared to non-completers. Control completers were significantly less variable in stance-time, step velocity, and step length than non-completers (Supplementary Table 4).

### Progression of Gait Impairment Over 72 Months

**Figure 2:**
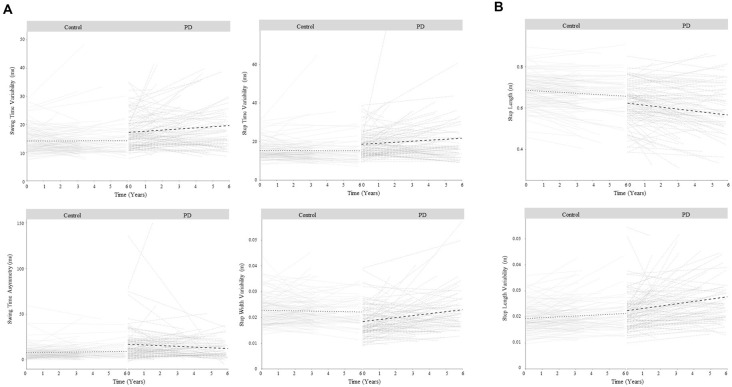
Change in gait over 6 years for control and Parkinson’s disease (PD) participants for gait characteristics that demonstrated a significant change in PD related to disease progression. Panel **(A)** shows gait characteristics that significantly changed in PD but not controls so demonstrated change related to disease progression only. Panel **(B)** shows gait characteristics that significantly changed in PD and control groups, but the rate of change was greater in PD, so demonstrated change related to both aging and disease progression. Bold lines show overall change within each group; faint lines show change for individuals.

In both groups, gait characteristics from all five domains significantly progressed over 72 months. Progression was more extensive in the PD cohort. In basic change models, ten of sixteen characteristics significantly changed in PD compared to seven characteristics in controls (Table 2, Supplementary Figure 1). In PD, gait became significantly slower, with a shorter step length, greater variability (swing and step time, step length, step width), reduced asymmetry (swing time), wider step width, and faster steps (step and swing time). Gait also became significantly slower in controls, with shorter steps, greater variability (step length), greater asymmetry (step length), wider step width, and faster swing time. Variation was considerable between individuals in the trajectories of change for each gait characteristic (Figure 2). The overall severity of PD-related motor symptoms also worsened as shown by a 3.0 point increase per year in the UPDRS III (CI_95%_ 2.51, 3.61, *p* < 0.001).

### Discerning PD-Specific Gait Changes From Aging

**Figure 3:**
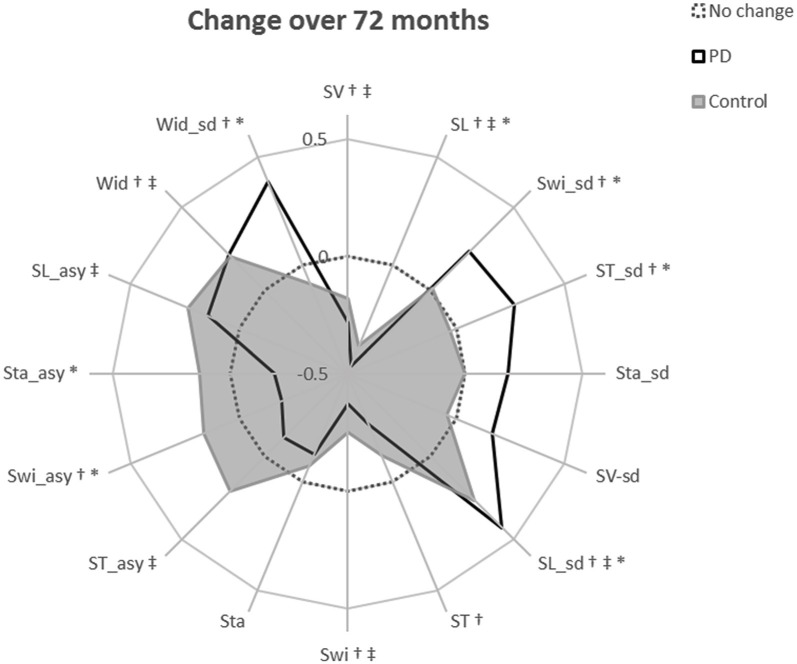
Radar plot illustrating the relative change in each gait characteristic over 72-months. The central dotted line represents no change. Deviations from zero along the axes radiating from the center of the plot represent the relative change in each gait characteristic over 72-months within each diagnostic group, calculated as the modeled change per year divided by the standard deviation of the modeled change. Gait characteristics are abbreviated as follows: SV, step velocity; SL, step length; Swi, swing time; ST, Step time; Sta, Stance time; Wid, Step width; sd, standard deviation (gait variability); asy, asymmetry. ^†^indicates a significant change in gait in the PD cohort over 72 months; ^‡^indicates a significant change in gait in controls over 72 months; *indicates a significantly different rate of change in PD gait compared to the rate of gait change in controls. ^†^*Denotes criteria 1 satisfied; ^†^^‡^denotes criteria 2 satisfied; ^†^^‡^*denotes criteria 3 satisfied.

In line with the second study aim, the ten significant gait changes identified within the PD cohort have been separated into those relating to aging and/or disease progression, according to three criteria outlined in the methods. Four characteristics demonstrated change-related only to disease progression, meeting criterion 1. Specifically, increasing variability (step and swing time), reducing asymmetry (swing time), and worsening postural control of gait (increasing step width variability) were significant changes in PD but not controls (Figure 2A), and change differed significantly between the groups (significant Group × Time interaction, Table 2, Figure 3).

Three characteristics changed solely due to aging, meeting criteria 2: slowing step velocity, quickening swing time, and increasing step width were exhibited in PD and controls at similar rates. Step time changed in the PD group only, but does not meet any outlined criteria so has not been considered further.

Finally, two characteristics demonstrated change related to both aging and disease progression, meeting criteria 3. Step length shortened and step length variability increased significantly in both groups; however, the rate of change was significantly greater in PD than controls (Figure 2B).

### Associations Between Gait Changes and Change in Levodopa Dose Over Time

LEDD increased by 106 mg/day each year. Ninety-three percent of people with PD had increased LEDD compared to baseline assessment (two had a decreased dosage, six remained at baseline dosage). Only one gait characteristic was related to LEDD change; larger increases in step width variability related to greater increases in LEDD over time (Table 2). Inclusion of the LEDD × Time interaction resulted in no significant change in step width variability (*p* > 0.05), indicating that step width variability change is at least partially explained by a change in LEDD. All other gait characteristics meeting criteria 1 and 3 did not show associations between gait change and LEDD change and therefore exhibited disease-specific change that was not related to levodopa adjustments (Figure 3, Supplementary Figure 1).

## Discussion

This is the first study to objectively and quantitatively model progression of gait impairment over 6 years in an incident cohort of PD compared to a well-matched control group. This demonstrated changes associated primarily with aging rather than PD progression, and also those characteristics due to aging which are accelerated by the presence of PD. In people with PD, we identified four gait characteristics that showed disease-specific progression, three that showed age-related progression, and two that could be explained through a combination of age and disease-specific change. Most gait changes occurred irrespective of increasing dopaminergic medication, highlighting limitations of dopaminergic therapies, and indicating the involvement of other mechanisms of gait impairment. The broad lack of correlation between increases in levodopa and gait decline indicates a significant variation in how gait impairments respond to dopaminergic therapy between people with PD. Novel therapies targeting non-dopaminergic mechanisms are therefore required to slow the progression of gait progression and reduce the associated consequences of falls and reduced activity. Ultimately, these findings improve the current understanding of mechanisms of gait impairment in early PD.

### Changes in Gait Over 6 Years

Our first aim was to describe gait changes in PD and controls over an extended period of 6 years. As expected, many gait characteristics changed in both PD and control cohorts; changes in gait occurred more frequently and at greater magnitudes in PD compared to changes in controls. Several changes were unique to each cohort, highlighting the specificity of gait change.

### PD-Specific Changes in Gait

To fulfill our second aim, three criteria established gait changes specific to PD pathology (criteria 1), reflective of aging (criterion 2), or indicative of a combination of PD progression and aging (criteria 3). Four characteristics significantly changed in PD only (increased variability of swing time, step time and step width, and reduced swing time asymmetry) and describe changes specifically due to disease progression. These findings extend previous work in this cohort over shorter periods (Galna et al., 2015; Rochester et al., 2017; Hobert et al., 2019) by revealing additional changes in asymmetry and variability that are not evident over 18 or 36 months. Our findings are also supported by a smaller study that showed step time variability increased more over 5 years in 22 people in the early stages of PD compared to a typically aging cohort (Hobert et al., 2019). We were not able to confirm the specific disease mechanisms underlying changes in variability (step, swing, step width) in this study. However, evidence suggests that increasing variability may be due to aggregation of disease pathologies over the first 6 years of PD affecting the neural control of gait, including amyloid and tau (Kang et al., 2013; Rochester et al., 2017). Whilst these analyses using cerebrospinal fluid (CSF) markers cannot inform us of the specific neural regions where pathological aggregates may most affect gait variability, assessments of amyloid with gait speed indicate that greater pathological burden within basal ganglia regions, the temporal cortex, and the anterior cingulate may be associated with worse gait performance in healthy older adults (Del Campo et al., 2016; Nadkarni et al., 2017; Wennberg et al., 2017). Neurological changes exacerbated by PD may additionally influence changes in gait variability (Wilson et al., 2019). Greater gait asymmetry in people with PD at baseline is in agreement with an asymmetrical pattern of neuronal loss (Wang et al., 2015; Claassen et al., 2016). Moreover, reduced asymmetry over time in PD likely relates to bilateral neuronal loss with PD progression (Rousseaux et al., 2012). Further work is needed to define these mechanisms more clearly.

### The Role of Levodopa in PD Gait Progression

To further understand the drivers of gait progression specific to PD, we explored the relationship between change in gait and change in levodopa, addressing our third aim. This study, along with previous work (Rochester et al., 2017), demonstrated that increasing step width variability is related to increasing levodopa medication. The reasons for this are unclear and the increase in step width variability in response to levodopa medication can be interpreted in different ways. Speculatively, increased levodopa may allow for more precise control of mediolateral foot placement. In contrast, a more variable step width may be a compensation for balance difficulties aggravated by increased levodopa dosage (Curtze et al., 2015). It is also possible that changes in levodopa and step width variability both reflect a more progressive PD phenotype and are not causally linked. No other gait characteristics were related to levodopa change. Dopaminergic medication may therefore mitigate the pathophysiological progression of gait impairment to an insufficient extent, or the progression of discrete gait impairments may be resistant to levodopa medication (dopa-resistant). Also, dopaminergic medications are most commonly used to treat motor symptoms in PD yet their extended use may have a negative effect on gait. Whilst the severity of freezing of gait (FOG) is thought to lessen with dopaminergic medications, it has been reported that FOG may be less prevalent in those not treated with levodopa (Garcia-Ruiz, 2011; Koehler et al., 2019; Nonnekes et al., 2020). Additionally, the use of dopamine agonists, which augment the effects of levodopa, may lead to an increased risk of falling or induce FOG (Serrao et al., 2015). Furthermore, the effects of dopaminergic medications lessen with disease progression (Jenner, 2015) and long-term use of dopaminergic therapies typically leads to involuntary movements known as dyskinesia (Iravani and Jenner, 2011). Changes in gait reported in this study may have therefore been augmented by continued levodopa usage; to understand this further, gait progression could be monitored in levodopa-naïve PD participants in the future and compared to findings presented here. These challenges highlight the need for novel non-dopaminergic therapies (Curtze et al., 2015; Galna et al., 2015).

Although the precise mechanisms of non-dopaminergic gait control are unclear, emerging evidence suggests the importance of the cholinergic system (Morris et al., 2019). Cholinergic neurons in the pedunculopontine nucleus (PPN) influence gait and postural control (Karachi et al., 2010), and slower walking speed in PD is associated with increases in short-latency afferent inhibition (Rochester et al., 2012) and cholinergic denervation (Bohnen et al., 2013; Müller et al., 2015). Also, deep brain stimulation (DBS) within the PPN may improve step velocity (Thevathasan et al., 2012); suggesting interventions that target brain regions not primarily dependent on dopamine may therefore help to mitigate gait impairment in PD. The benefits of drugs targeting the cholinergic system on PD gait are also being explored (Henderson et al., 2016; Smulders et al., 2016; Müller et al., 2019). Overall, interpretation of the relationship between dopamine and gait progression is limited as gait was not assessed “off” medication, nor were biomarkers of dopaminergic activity such as DAT imaging used. Nevertheless, our findings indicate that discrete gait characteristics progress irrespective of levodopa, suggesting the importance of non-dopaminergic mechanisms in gait impairment.

### The Contribution of Aging to PD Gait Progression

Understanding how gait changes with normal aging is important as age-related changes cumulatively contribute to gait progression in people with PD alongside disease progression. Three characteristics changed in both PD and control cohorts over 6 years; the change was therefore considered to be due primarily to aging mechanisms rather than disease progression. These changes (slowing of gait, widening of steps, and quickening swing time) met criteria 2.

Slowing of gait is considered a typical feature of PD progression. In this study, the walking speed of people with PD slowed by 1.24 cm/s per year (CI_95%_: 0.4 to 2.1 cm/s per year). This is not as severe a decline as reported by either Ellis et al. (2016; 4 cm/s per year) or Hobert et al. (2016; 3.4 cm/s per year) in early PD, yet faster than the 4.3 cm/s per year increase in walking speed during a 6-min walk reported by Cavanaugh et al. (2012). Differences in demographic characteristics, disease severity and duration, testing setting and paradigm, and treatment regime may all help explain differences in the mean estimate of change between these studies. Moreover, each of these studies reported a large variation in change over time between participants, indicating that gait impairment evolves differently for each individual with PD, warranting much-needed research for prognostic markers of gait decline in PD to better inform personalized treatment. Interestingly, we also found that walking speed (step velocity) slowed over time in both groups, suggesting this is a more general feature of aging. Although the rate of change was greater in PD this was not significant, replicating previous findings (Galna et al., 2015; Hobert et al., 2019). While walking speed may be a useful measure of change and response to therapy, interpreting change in walking speed is more complex and we posit are more likely to reflect a combination of aging and disease. This may explain, in part, the large inter-individual variation in rates of gait change within each group. Walking speed provides an overall measure of gait performance, influenced by discrete characteristics such as step length, timings of steps, and step width (Wade, 1992; Galna et al., 2015). Further investigation into predictive factors for gait change in PD and healthy aging cohorts may give better insight into the reasons behind individual differences in gait progression and aid better interpretation of reduced gait speed in PD and aging cohorts. Small yet clinically meaningful changes in gait speed are reported as 3-6 cm/s in older adults with mobility issues (Perera et al., 2006) and in PD (Hass et al., 2014). Our work, therefore, indicates that a clinically meaningful change in gait typically occurs within the first 6 years of PD (7.4 cm/s over 6 years in people with PD; see Table 2 for estimates of yearly change). Overall, given that change in step velocity does not significantly differ between PD and control groups, looking beyond walking speed is important in order to allow more nuanced interpretation of changes and their underlying mechanisms.

Age-related change contributes to gait impairments observed in PD, suggesting that therapies addressing features of aging may be effective in reducing the burden of PD. It is important, therefore, to consider the mechanisms driving age-related gait change. Change may be due to atrophy and loss of muscle strength (Abernethy, 2005; Song and Geyer, 2018), physical inactivity (Busch et al., 2015), and development of age-related conditions, such as osteoarthritis (Zhang and Jordan, 2010) causing increased pain and stiffness during movement (Jayakody et al., 2018). Age-related changes in the brain such as atrophy and white matter abnormalities also explain the slowing and increased variability of gait during aging (Wilson et al., 2019). Increasing evidence implies that specific neural regions or networks underpin discrete gait characteristics (Tian et al., 2017), which could be specifically targeted to prevent discrete components of age-related gait decline. On this point, gait performance in PD is improved by exercise-based interventions aiming to increase muscle strength and activity (van der Kolk and King, 2013; Shen et al., 2016); speculatively these therapies may, at least partially, be targeting age-related changes which in turn positively impact PD gait.

Two gait characteristics, step length, and step length variability, significantly worsened within PD and control groups over 6 years and specifically, those changes occurred more rapidly in PD. These characteristics met criteria 3, suggesting that both PD and age-related mechanisms contributed to their progression. In previous work over a shorter time frame [36 months from diagnosis (Rochester et al., 2017)] step length change was not significantly different in PD compared to controls. This may be due to shorter step length in PD strongly associating with dopaminergic dysfunction (Galna et al., 2015) and the resultant hypokinesia (Morris et al., 1996), so may decline as dopaminergic medications become less effective over time due to neuronal cell loss (Zahoor et al., 2018). Alternatively, change may simply be due to loss of muscle mass and strength over time (sarcopenia), as a result of aging or as a secondary consequence of the reduced activity observed during PD. Further investigation into the underlying mechanisms of these gait changes such as direct measurement of muscle mass over time may indicate whether these are purely age-related changes accelerated by disease progression, or whether aging and disease mechanisms act independently of each other to cause change. Collectively, our findings highlight the benefits of longer follow-up duration, through identifying changes related specifically to disease progression and changes reflective of both disease progression and aging over 6 years. These gait characteristics may act as new therapeutic targets for the prevention of gait progression in PD.

### Study Strengths and Limitations

This study is the largest to document gait change in PD over the longest period from diagnosis, in a relatively homogeneous cohort of incident PD participants. The main strength was that gait changes due to aging and disease progression could be parsed, as a well-matched control cohort was assessed alongside people with PD. Relatively precise modeling of gait change in early PD was achieved as PD participants were recruited close to diagnosis, enabling monitoring over the first 6 years of the disease. Inter-individual variation was accounted for through the random effect term included in all models. The inclusion of LEDD in analyses enabled the investigation of gait changes that were related and, arguably, more importantly, not related to changes in dopaminergic medication, to identify potential therapeutic targets for non-dopaminergic interventions. The effects of dopamine on gait progression could be further explored by comparing gait progression for characteristics measured “on” and “off” medication. Specifically, testing participants both on and off medication will allow us to better understand the progression of the underlying disease as well as explore the complex relationship between step width variability and dopaminergic medication.

Some limitations should be noted. As with many longitudinal studies, attrition was substantial (57%) although comparable to similar studies. PD completers were younger, with a less severe motor disease, better global cognition, and less severe gait impairment at baseline, indicating a potential underestimation of overall progression rates. Mixed-effects modeling allowed the use of all data, reducing bias compared to traditional analytical approaches. PD misdiagnosis is unlikely to have affected findings as the diagnosis was reviewed at each assessment and the number of revised diagnoses was low (Figure 1). We chose not to correct for multiple comparisons; this was justified as it allowed for direct comparison with gait changes over 36 months (Rochester et al., 2017), and reduced the risk of type II error. To account for the possibility of type I errors, we have been cautious in our interpretation of the findings and also provided *p*-values for each comparison for transparency. Finally, it was beyond the scope of the aims of this article to examine underlying non-dopaminergic and cognitive mechanisms of gait decline in people with PD, however, these remain pertinent topics for future research.

## Conclusions

Discrete gait impairments progress over 6 years after a diagnosis of PD, due to a combination of disease-specific and age-related mechanisms. Gait changes were mostly unrelated to dopaminergic medication adjustments, highlighting limitations of current dopaminergic therapy and the need to improve interventions targeting gait decline in PD.

## Data Availability Statement

The raw data supporting the conclusions of this article will be made available by the authors, without undue reservation.

## Ethics Statement

The studies involving human participants were reviewed and approved by Newcastle and North Tyneside research ethics committee, Tyen and Wear, United Kingdom. The patients/participants provided their written informed consent to participate in this study.

## Author Contributions

JW: statistical analysis: design and execution; manuscript preparation: writing the first draft, review and critique. LA: research project: execution; statistical analysis: review and critique; manuscript preparation: review and critique. AY: research project: execution; manuscript preparation: review and critique. SL: research project: organization and execution; manuscript preparation: review and critique. RL: research project: execution; statistical analysis: review and critique; manuscript preparation: review and critique. RM: research project: execution; manuscript preparation: review and critique. J-PT: statistical analysis: review and critique; manuscript preparation: review and critique. DB: research project: conception; manuscript preparation: review and critique. LR: research project: conception, organization and execution; statistical analysis: review and critique; manuscript preparation: review and critique. BG: research project: execution; statistical analysis: design, review, and critique; manuscript preparation: review and critique. All authors contributed to the article and approved the submitted version.

## Conflict of Interest

AY is supported by the Newcastle NIHR Biomedical Research Centre. She has received honoraria from Teva-Lundbeck and sponsorship from Teva-Lundbeck, UCB, GlaxoSmithKline, Genus, Britannia, and AbbVie for attending conferences. Her research is supported in part by grants from Parkinson’s UK, the National Institute for Health Research, European Union, Michael J. Fox Foundation and Dunhill Medical Trust. LR’s research program is supported in part by grants from the Medical Research Council (MRC), European Union, Parkinson’s UK, and the National Institute for Health Research Biomedical Research Unit (NIHR BRU) for Lewy Body Dementias. DB is supported by grants from Parkinson’s UK, National Institute for Health Research Health Technology Assessment (NIHR HTA), MRC, and Newcastle Healthcare Charity and has received consultancy from the Michael J. Fox Foundation and honorarium for lectures from Teva-Lundbeck and Union Chimique Belge (UCB). J-PT is supported by National Institute for Health Research (NIHR) Newcastle Biomedical Research Centre (BRC) based at Newcastle upon Tyne Hospitals NHS Foundation Trust and Newcastle University. RL is supported by grants from Parkinson’s UK. JW is supported by grants from the Wellcome Trust (UNS35604). The remaining authors declare that the research was conducted in the absence of any commercial or financial relationships that could be construed as a potential conflict of interest.
