# Supplementary material for: Gait Progression Over 6 Years in Parkinson’s Disease: Effects of Age, Medication, and Pathology
Source: Front Aging Neurosci. 2020 Oct 15;12:577435. doi: 10.3389/fnagi.2020.577435 (PMC7593770; doi:10.3389/fnagi.2020.577435)
Supplement: Supplementary file 1 [file Data_Sheet_1.PDF]

Supplementary Table 1 The number of participants that completed assessments up until 72 months

Total number of assessments	Time points at which assessments took place					PD frequency	Control frequency
	Baseline	18 months	36 months	54 months	72 months		
**1**	●					10	54
**2**	●	●				24	10
	●		●			1	56
	●			●		1	0
**3**	●	●	●			18	3
	●	●		●		0	1
	●	●			●	1	0
**4**	●	●	●	●		13	7
	●	●	●		●	2	1
	●	●		●	●	2	1
	●		●	●	●	1	2
**5**	●	●	●	●	●	46	49
						**119**	**184**

Supplementary Table 2 Descriptive and clinical characteristics of PD and control "completers" and "non-completers", presented as mean (sd).

Demographic or clinical characteristic	Control			PD		
	**Completers (n = 53)**	**Non-completers (n=77)**	**Group difference *(test statistic, p)***	**Completers (n = 52)**	**Non-completers (n=57)**	**Group difference *(test statistic, p)***
Age (years)	67.0 (6.3)	71.3 (7.6)	**t=3.41, p=.001***	64.8 (9.8)	69.7 (9.5)	**t=2.63, p=.010***
Sex	25f 28m	47f 30m	χ^2 =2.44, p=.118	17f 35m	20f 37m	χ^2 =0.07, p=.792
Height (m)	1.71 (0.09)	1.66 (0.10)	**t=-2.73, p=.007***	1.70 (0.08)	1.70 (0.08)	t=0.05, p=.959
Body mass (kg)	82.7 (14.8)	75.3 (14.3)	**t=-2.84, p=.005***	77.5 (13.4)	80.0 (17.0)	t=0.85, p=.396
GDS	0.9 (1.1)	1.5 (2.4)	U=1825, p=.274	2.4 (2.1)	2.7 (2.3)	U=1356, p=.436
Education (years)	14 (4)	12 (3)	U=411, p=.077	14 (4)	13 (4)	U=1265, p=.184
NART	117 (8.3)	117 (7.3)	U=2024, p=.936	116 (11)	114 (11)	U=1297, p=.333
MoCA	27.5 (2.2)	26.0 (2.4)	**U=341, p=.012***	26.2 (3.1)	24.2 (3.8)	**U=921, p=.005***
MMSE	29.3 (1.0)	29.2 (1.1)	U=1184, p=.409	28.9 (1.1)	28.5 (1.3)	U=1224, p=.103
Sit to stand (s)	12.1 (3.0)	12.2 (3.7)	U=1967, p=.820	13.5 (4.3)	14.5 (5.0)	U=1197, p=.325
Single leg stance (s)	16.9 (11.3)	15.7 (11.6)	U=1887, p=.463	17.0 (10.7)	10.9 (10.1)	**U=956, p=0.001***
ABC (0-100)	92.6 (11.5)	92.8 (8.0)	U=1887, p=.467	87.4 (16.7)	77.5 (20.7)	**U=1093, p=.018***
MDS-UPDRS III (0-132)	-	-	-	22.1 (9.3)	27.7 (10.5)	**t=2.96, p=.004***
Hoehn and Yahr stage n (%)	-	-	-	I 18 (35%); II 26 (50%); III 8 (15%)	I 8 (14%); II 39 (68%); III 10 (18%)	**χ^2 =6.45, p=.040***
n (%) who report FoG	-	-	-	5 (9.6%)	6 (10.5%)	χ^2 =0.03, p=.875
LEDD (mg/day)	-	-	-	160 (117)	190 (144)	t=1.18, p=.241

Supplementary Table 3. Differences in baseline gait between PD and control, presented as mean (sd). Descriptives of untransformed variables are presented here.

Gait characteristic	Control (n = 130)	PD (n=109)	Group difference *(p)*
Step velocity (m/s)	1.29 (0.17)	1.13 (0.21)	**<0.001***
Step length (m)	0.69 (0.07)	0.62 (0.10)	**<0.001***
Swing time variability (ms)	14.34 (4.15)	17.30 (5.81)	**<0.001***
Step time variability (ms)	15.37 (4.46)	18.53 (6.37)	**<0.001***
Stance time variability (ms)	18.33 (6.06)	22.53 (9.27)	**<0.001***
Step velocity variability (m/s)	0.05 (0.01)	0.05 (0.02)	.465
Step length variability (m)	0.02 (0.01)	0.02 (0.01)	**<0.001***
Step time (ms)	533.80 (45.21)	556.00 (45.10)	**<0.001***
Swing time (ms)	385.54 (30.53)	389.37 (32.14)	.347
Stance time (ms)	682.65 (66.46)	723.05 (71.79)	**<0.001***
Step time asymmetry (ms)	9.78 (8.17)	20.57 (25.40)	**<0.001***
Swing time asymmetry (ms)	8.00 (7.84)	16.96 (20.00)	**<0.001***
Stance time asymmetry (ms)	7.96 (8.12)	16.63 (19.52)	**<0.001***
Step length asymmetry (m)	0.02 (0.02)	0.03 (0.02)	**.007***
Step width (m)	0.09 (0.02)	0.09 (0.03)	.279
Step width variability (m)	0.02 (0.01)	0.02 (0.01)	**<0.001***

Supplementary Table 4 Gait characteristics for PD and control "completers" and "non-completers", presented as mean (sd). Descriptives of untransformed variables are presented here.

**Gait characteristic**	**Control**			**PD**		
	**Completers (n = 53)**	**Non-completers (n=77)**	**Group difference *(p)***	**Completers (n = 52)**	**Non-completers (n=57)**	**Group difference *(p)***
Step velocity (m/s)	1.29 (0.14)	1.30 (0.18)	.953	1.19 (0.20)	1.08 (0.20)	**.006***
Step length (m)	0.69 (0.07)	0.68 (0.08)	.319	0.65 (0.10)	0.59 (0.09)	**.001***
Swing time variability (ms)	13.56 (3.78)	14.87 (4.34)	.064	16.01 (4.34)	18.47 (6.71)	.053
Step time variability (ms)	14.66 (4.23)	15.87 (4.58)	.099	17.64 (5.87)	19.33 (6.74)	.170
Stance time variability (ms)	17.12 (5.47)	19.16 (6.33)	**.039***	21.49 (9.05)	23.48 (9.44)	.209
Step velocity variability (m/s)	0.05 (0.01)	0.05 (0.01)	**.002***	0.05 (0.02)	0.05 (0.02)	.741
Step length variability (m)	0.02 (<0.01)	0.02 (0.01)	**.015***	0.02 (0.01)	0.02 (0.01)	.133
Step time (ms)	539.42 (43.72)	529.94 (46.09)	.241	556.38 (45.07)	555.64 (45.53)	.933
Swing time (ms)	385.32 (30.48)	385.69 (30.77)	.947	394.82 (31.74)	384.40 (31.97)	.091
Stance time (ms)	694.56 (63.92)	674.44 (67.35)	.090	718.23 (70.53)	727.43 (73.28)	.507
Step time asymmetry (ms)	11.12 (8.46)	8.86 (7.88)	.084	22.89 (31.17)	18.45 (18.69)	.419
Swing time asymmetry (ms)	8.22 (7.31)	7.86 (8.22)	.723	17.54 (24.82)	16.43 (14.48)	.717
Stance time asymmetry (ms)	8.10 (8.11)	7.87 (8.18)	.946	17.69 (24.05)	15.66 (14.35)	.941
Step length asymmetry (m)	0.02 (0.02)	0.02 (0.01)	.976	0.03 (0.03)	0.02 (0.02)	.791
Step width (m)	0.09 (0.02)	0.09 (0.03)	.801	0.09 (0.03)	0.09 (0.03)	.488
Step width variability (m)	0.02 (0.01)	0.02 (0.01)	.331	0.02 (0.01)	0.02 (<0.01)	.285

Supplementary Figure 1 A summary of longitudinal analysis completed, indicating whether gait change was significant in each group or different between the groups, and whether change in levodopa medication was related to gait progression.

		Significantly changed over time in typical aging?	Significantly changed over time in PD?	Change over time different between PD and controls?	PD change over time related to levodopa dose change?
**Pace**	Step velocity	↘	↘	-	-
	Step length	↘	↘	✓	-
	Swing time variability	-	↗	✓	-
**Variability**	Step time variability	-	↗	✓	-
	Stance time variability	-	-	-	-
	Step velocity variability	-	-	-	-
	Step length variability	↗	↗	✓	-
**Rhythm**	Step time	-	↘	-	-
	Swing time	↘	↘	-	-
	Stance time	-	-	-	-
**Asymmetry**	Step time asymmetry	↗	-	-	-
	Swing time asymmetry	-	↘	✓	-
	Stance time asymmetry	-	-	✓	-
**Postural Control**	Step length asymmetry	↗	-	-	-
	Step width	↗	↗	-	-
	Step width variability	-	↗	✓	↗

Notes: Arrows indicate the direction of association; more darkly shaded boxes indicate a stronger association (relative to associations made within each set of analysis).
